# Infectious Diseases Physicians' Attitudes and Practices Related to Complementary and Integrative Medicine: Results of a National Survey

**DOI:** 10.1155/2013/294381

**Published:** 2013-07-14

**Authors:** Kalpana D. Shere-Wolfe, Jon C. Tilburt, Chris D'Adamo, Brian Berman, Margaret A. Chesney

**Affiliations:** ^1^Department of Medicine, University of Maryland, 29 South Greene Street, Suite 300, Baltimore, MD 21201, USA; ^2^Department of Medicine, Mayo Clinic, 200 First Street SW, Rochester, MN 55905, USA; ^3^Department of Family and Community Medicine, University of Maryland, 520 W. Lombard Street, East Hall Room 204, Baltimore, MD 21201, USA; ^4^Osher Center for Integrative Medicine, University of California, 1545 Divisadero Street, San Francisco, CA 94143, USA

## Abstract

*Background*. Complementary and alternative medicine (CAM) and integrative medicine (IM) modalities are widely used by patients, including those with infectious diseases (ID). *Methods*. One thousand randomly selected ID practitioners were surveyed. The survey was divided into domains related to familiarity and recommendation, beliefs and attitudes, and use of CAM/IM modalities. *Results*. The response rate was 31%. ID physicians were most familiar with vitamin and mineral supplementation (83%), massage (80%), acupuncture (79%), chiropractic (77%), yoga (74%), and herbal medicine (72%). ID physicians most recommended vitamin and mineral supplementation (80%) and massage (62%). Yoga, meditation, and acupuncture were recommended by 52%, 45%, and 46%, respectively. Drug interactions, clinical research, and knowledge of CAM/IM modalities were factors that were considered a major influence. Almost 80% of respondents indicated an interest in IM versus 11% for CAM. Most respondents (75%) felt that IM modalities are useful, and more than 50% believed that they could directly affect the immune system or disease process. *Conclusion*. ID physicians expressed a markedly greater interest for IM versus CAM. They appear to be familiar and willing to recommend some CAM/IM modalities and see a role for these in the management of certain infectious diseases. Data regarding clinical efficacy and safety appear to be important factors.

## 1. Introduction

In the United States of America (USA), as defined by the National Center for complementary and Alternative Medicine (NCCAM), complementary and alternative medicine (CAM) is a group of diverse medical and health care systems, practices, and products that are not presently considered part of conventional medicine and integrative medicine is medicine that combines treatments from conventional medicine and CAM for which there is some high-quality evidence of safety and effectiveness [1]. Both of these terms are used in the United States. 

Both CAM and integrative medicine modalities are widely used by patients, including those with infectious diseases [2, 3]. Amongst human immunodeficiency virus (HIV)- infected patients, studies have shown that a large percentage uses these modalities [2, 4–6]. One review of 40 studies of CAM use in HIV-infected patients revealed approximately 60% report CAM use [3]. Other infectious diseases and associated conditions for which CAM and integrative medicine modalities have been used include common cold, recurrent urinary tract infections (UTI), malaria, diarrhea, and HIV/highly active antiretroviral therapy (HAART)- associated hypertriglyceridemia [7].

Several studies have assessed how general practitioners and some specialists view CAM and integrative medicine therapies [8–15]. However, there is little data evaluating how infectious diseases (ID) physicians view these modalities. In one study of 89 HIV care providers, 63% believed that CAM and integrative medicine therapies may be helpful for HIV-infected patients and 36% had personally used one [16]. There is no national data describing how general ID practitioners view these modalities. 

The purpose of this study was to determine how ID practitioners in the USA view CAM and integrative medicine modalities by defining the following. (1) How familiar are ID practitioners with CAM and integrative medicine modalities and which ones do they recommend? (2) What are the perceived obstacles to the use of these modalities? (3) What are ID physicians' beliefs and attitudes toward them and what role, if any, do they see for them in their patients?

## 2. Material and Methods

### 2.1. Study Population

One thousand self-identified ID practitioners in the USA were randomly selected from the 2010 American Medical Association master file. On June 15, 2010, a confidential, voluntary, and self-administered survey, cover letter, and laser pen were mailed to the selected recipients. A second mailing (September 14, 2010) included a revised survey and cover letter. For the third mailing (November 15, 2010), nonresponders were sent a paper, web-based survey, or both.

### 2.2. Ethical Review

This survey was declared exempt after review by the University of Maryland and Mayo Clinic Institutional Review Board. 

### 2.3. Survey Instrument

The survey was developed by drafting an instrument, in-depth sessions with ID physicians, and repeated revisions. The survey focused on ID practitioners' knowledge, beliefs, and use of CAM and integrative medicine modalities within the accepted major categories (displayed in Table 1). The final instrument was organized into 4 domains: (1) familiarity with and recommendation of CAM and integrative medicine modalities, (2) beliefs and attitudes towards CAM and integrative medicine modalities, (3) use of CAM and integrative medicine modalities in clinical practice,and (4) participant and practice characteristics. 

### 2.4. Survey Data

#### 2.4.1. Familiarity and Recommendation

To assess ID practitioners' familiarity with and recommendation of CAM and integrative medicine modalities, we asked the respondent to answer “yes” or “no” to the statement “I am familiar with this modality.” If the respondents indicated familiarity then they were asked to answer “yes” or “no” to the following statement: “If yes, I have recommended this modality.”

#### 2.4.2. Barriers to Use

To further explore what considerations or barriers to use are important to ID physicians we asked them to rate potential barriers as “major,” “minor,” or “not at all.” The items evaluated were (1) knowledge of how and when to use CAM and integrative medicine modalities, (2) amount of clinical research showing clear benefit, (3) insurance coverage, (4) cost, (5) reliable referral base, (6) concern for professional reputation, (7) fear of judgment by colleagues, (8) insufficient regulatory oversight of supplements, and (9) potential drug interactions with botanicals and supplements. 

#### 2.4.3. Beliefs and Attitudes ****

*Nomenclature. *To determine whether ID physicians have a preference for the term integrative medicine or complementary and alternative medicine, we provided participants definitions and asked which they would be more interested in learning about. The CAM definition was “a group of diverse medical and health care systems, practices, and products that are not presently considered to be part of conventional medicine” [1]. The integrative medicine definition was “the practice of medicine that reaffirms the importance of the relationship between practitioner and patient, focuses on the whole person, is informed by evidence, and makes use of all appropriate therapeutic approaches, health care professionals, and disciplines to achieve optimal health and healing” [17]. 

*Perceived Benefit. *To determine how ID physicians perceived benefit of specific modalities for specific medical conditions for which there is research evidence of benefit, participants were asked to evaluate the following therapeutic combinations as “beneficial,” “moderately beneficial,” “slightly beneficial,” or “insufficient knowledge”: (1) artemisinins for malaria, (2) cranberry for UTI, (3) zinc for diarrhea in malnourished children, and (4) omega-3 fatty acids for hypertriglyceridemia.

*Beliefs about CAM and Integrative Medicine Modalities. *To further explore beliefs about CAM and integrative medicine modalities, participants were asked their extent of agreement on a 4-point scale with following statements: in general, CAM and integrative medicine modalities (1) are not useful, (2) derive their benefit from placebo effect, (3) are useful primarily in alleviating symptoms related to diseases and/or medications, (4) affect the underlying disease process, and (5) directly affect the immune system. For respondents who agreed with statements 4 and 5, an additional question asked to which specific CAM and integrative medicine modalities they were referring. 

*Role for CAM and Integrative Medicine Modalities. *To determine ID practitioners' opinions about roles for CAM and integrative medicine modalities in infectious diseases, we inquired about the potential use of them in the overall treatment plan of HIV and HIV-related complications, acute bacterial infections, hepatitis, chronic Lyme disease, autoimmune complications of an infectious disease, and recurrent bacterial infections. Finally we sought to determine which of the following considerations was the most important factor for determining patient treatment by asking them to choose from the following: (1) data based on clinical research, (2) patient preference, (3) clinical response or benefit, and (4) clinical judgment. 

#### 2.4.4. Patient Characteristics

The following demographic and practice information was reported by the respondents: age group, race, sex, type of degree, board certification, practice setting, affiliation with a CAM and integrative medicine center, number of years in practice, category of work (research, patient care, teaching, administration, public health, and other), and proportion of time spent in various types of clinical work (HIV, hepatitis, chronic recurrent infections, hospital-acquired infections, outpatient care, infectious diseases, and general internal medicine). Additional demographic information for both responders and nonresponders, obtained through the AMA mailing list, included date of birth, region, place of birth, board certification, years in practice, age, sex, and type of practice. 

### 2.5. Statistical Analysis

Descriptive statistics were calculated for all survey items. Pearson's chi-square test was used to determine both the bivariate associations between participant characteristics and familiarity with and recommendation of CAM and integrative medicine modalities as well as in the sensitivity analyses assessing potential differences between survey responders and nonresponders. Multivariable logistic regression models were constructed to assess predictors of familiarity with and recommendation of CAM and integrative medicine modalities while simultaneously adjusting for all other potential predictors of interest. A two-sided *P* value of ≤0.05 was used to determine statistical significance of all associations. All analyses were performed using SAS version 9.1 (SAS Institute Inc., Cary, NC, USA).

## 3. Results

### 3.1. Demographics and Practice Characteristics

Approximately 31% (*n* = 311) of the ID practitioners surveyed responded to the survey. Demographic and practice characteristics are summarized in Table 2. There was no significant difference between responder and nonresponders with respect to sex, region, country of birth, degree, or board certification. There was a significant difference between the two groups with respect to transition year (*P* = 0.04) and type of practice (*P* = 0.007). There was a trend toward significance between the two groups based on age (*P* = 0.08). 

### 3.2. Familiarity and Recommendation

The familiarity and recommendations responses are displayed in Table 3. Overall, ID physicians were most familiar with and also most recommended manipulative and body-based modalities, biologically based modalities, and mind-body-based modalities. 

With respect to individual modalities, ID physicians were most familiar with vitamin and mineral supplementation (83%), massage (80%), acupuncture (79%), chiropractic (77%), yoga (74%), and herbal medicine (72%). They were least familiar with qi gong (17%), ayurveda (26%), and healing touch/reiki/therapeutic touch (39%). Of the ID physicians who were familiar with CAM and integrative medicine modalities, most recommended vitamin and mineral supplementation (80%) and massage (62%). Yoga, meditation, and acupuncture were recommended by 52%, 45%, and 46%, respectively. Respondents were least likely to recommend qi gong (6%) and homeopathy (8%). 

The greatest discrepancy between familiarity of a modality and the willingness to recommend it was for whole medical systems and energy-based modalities (ayurveda (86%), homeopathy (92%), traditional Chinese medicine (TCM) (89%), healing touch, reiki, therapeutic touch (83%), and qi gong (90%)). For mind-body-based modalities, ID practitioners were least likely to recommend hypnosis/guided imagery or tai chi despite familiarity with these modalities. For biologically based modalities, of those familiar with herbal medicine, 68% did not recommend it. Similarly, of those who were familiar with chiropractic, 68% did not recommend it. 

### 3.3. Barriers to Use

Respondents identified several factors that were considered a major influence on the use of CAM and integrative medicine modalities (Table 4). These were drug interactions with botanicals and supplements (82%), clinical research showing clear benefit (80%), and knowledge of CAM and integrative medicine modalities (72%).

### 3.4. Beliefs

#### 3.4.1. Nomenclature

Almost 80% of respondents indicated an interest in integrative medicine versus 11% for CAM. Another 11% indicated interest in neither.

#### 3.4.2. Perceived Benefit

**Figure 1:**
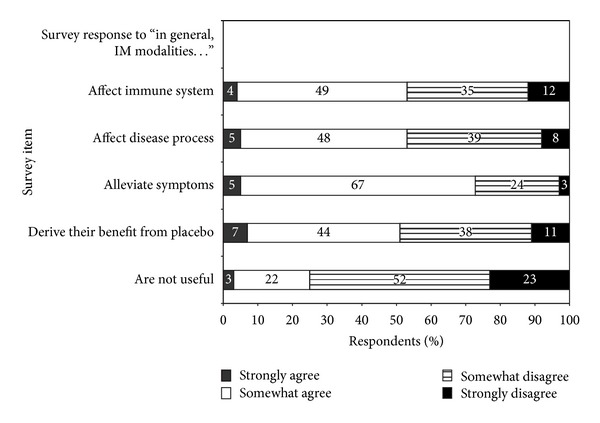
ID physicians' beliefs regarding CAM and integrative medicine modalities.

ID physicians' beliefs about CAM and integrative medicine modalities are summarized in Figure 1. In general, most respondents (75%) felt that CAM and integrative medicine modalities are useful. About half believed that CAM and integrative medicine modalities derive their benefit from placebo effect. Although a large percentage of respondents felt that CAM and integrative medicine modalities are useful for alleviating symptoms (73%), more than 50% (*n* = 157) also believed that one or more CAM and integrative medicine modalities could directly affect the immune system or the disease process: mind-body modalities (*n* = 90), botanicals/supplements (*n* = 96), manipulative and body-based medicine (*n* = 63), energy medicine (*n* = 24), and whole medical systems (*n* = 35).

**Figure 2:**
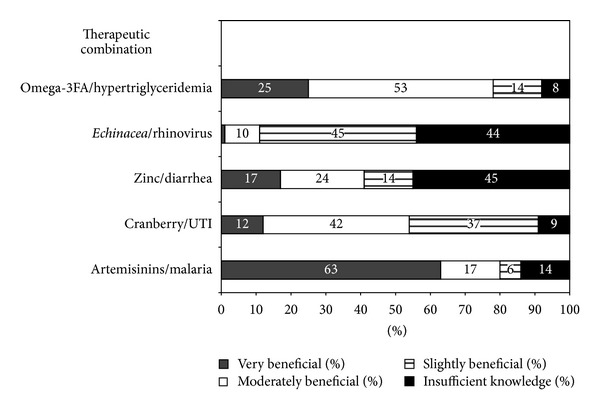
Perceived benefits of specific CAM and integrative medicine modalities for selected infectious diseases and associated conditions.

ID physicians' perceived benefit of specific CAM and integrative medicine modalities for specific infectious processes is displayed in Figure 2. The majority of ID physicians felt that artemisinins for malaria treatment and omega-3 fatty acids for the treatment of HIV-associated hyperlipidemia were beneficial. Approximately 50% felt that cranberry was beneficial for prevention of recurrent UTI. Only 10% felt that *Echinacea* was beneficial for the prevention of *rhinovirus infections*. Forty-five of respondents reported insufficient knowledge for the benefit of zinc and *Echinacea*. 

*Role for CAM and Integrative Medicine Modalities in ID. *Conditions for which ID physicians believed CAM and integrative medicine modalities could have a role in the overall treatment plan of a patient included HIV and HIV-related complications (*n* = 191), recurrent bacterial infections (*n* = 138), autoimmune complications (*n* = 121), “chronic Lyme disease” (*n* = 116), hepatitis B, C (*n* = 74), acute bacterial infections (*n* = 35), and others (*n* = 43).

Finally, the majority of respondents reported data based on clinical research as the most important factor in determining a treatment plan for their patients (68%), followed by clinical response or benefit (15%), clinical judgment (13%), and patient preferences (4%). 

### 3.5. Analysis

Neither the bivariate analysis nor the multivariate logistic regression modeling revealed any significant associations (*P* < 0.05) between the demographic and practice characteristics of the participants and familiarity with or recommendation of any of the CAM and integrative medicine modalities. 

## 4. Discussion

This is the first national study assessing the beliefs and practices of ID practitioners with respect to complementary, alternative, and integrative medicine modalities. The results reveal several notable findings. Many ID practitioners displayed both familiarity and a willingness to recommend CAM and integrative medicine modalities, particularly those in the manipulative and body-based, biologically based, and mind-body based categories. They were least familiar with and also least likely to recommend energy based modalities and whole medical systems modalities. Despite moderate familiarity with CAM and integrative medicine modalities such as homeopathy and traditional Chinese medicine, ID physicians were not willing to recommend them. These data raise important questions related to what factors influence the acceptance and integration of particular CAM and integrative medicine modalities within infectious diseases and also how this compares with general medical and other subspecialty fields. 

Surveys of general medical practitioners show varying rates of familiarity and recommendation. In one review of 25 physician surveys, physician referral rates ranged from highs of 83% for chiropractic and 71% for acupuncture to lows of 2% for chiropractic and 1% for homeopathy. The mean rates of referral for chiropractic, acupuncture, homeopathy, herbal medicine, and massage were 40%, 43%, 15%, 4%, and 21% [8]. The opinions among medical specialists also vary. In a survey of rheumatologists practicing in the USA, greater than 50% reported that chiropractic, acupuncture, mind-body practices, and body work were either very or moderately beneficial. In addition, greater than 50% of respondents were either very or somewhat likely to recommend acupuncture, mind-body practices, glucosamine +/− chondroitin, and bodywork [10]. In another study of Australian rehabilitation physicians, 80% reported familiarity with acupuncture, 74% with yoga, and 72% with tai chi [13].

In our study, ID physicians demonstrated high rates of familiarity with yoga (74%), meditation (69%), herbal medicine (72%), vitamin/minerals (83%), acupuncture (79%), massage (80%), and chiropractic (77%). They also demonstrated high rates of recommendation for vitamin/mineral therapy (80%), yoga (52%), meditation (45%), and massage (62%). Similar to other physicians, ID physicians reported lower familiarity and recommendation rates for energy-based medicine and whole medical systems [10, 13, 18, 19].

We found that despite familiarity with certain modalities, ID physicians were not willing to recommend them. In our study this was true for traditional Chinese medicine, homeopathy, healing touch, reiki, therapeutic touch, herbal medicine, chiropractic, and tai chi. Previous studies have shown that personal lack of knowledge, lack of data from clinical research, and referral base were important barriers to CAM referrals [20, 21]. In our study, we found that potential drug interactions, clinical research, knowledge, and regulatory oversight of botanicals and supplements were all important determinants in the use of CAM and integrative medicine modalities by ID physicians.

Both terms “Integrative Medicine” and “CAM” are used in the USA and we were interested in ascertaining if there was a preference for one over the other amongst ID physicians. The CAM definition we used was obtained from the National Center for Complementary and Alternative Medicine (NCCAM) website in 2010. NCCAM is the United States Federal Government's lead agency for scientific research on CAM [1]. The definition used for integrative medicine was adopted from The Consortium of Academic Health Centers for integrative medicine (CAHCIM) which includes 56 academic medical centers and affiliate institutions (in the USA, Canada, and Mexico) whose mission is to advance the principles and practices of integrative healthcare within academic institutions and their affiliates [17].

In this study, ID physicians in the United States demonstrated a clear preference for the term “Integrative Medicine” over “complementary and alternative medicine”. This preference may reflect their willingness to be open to therapies that are integrated into their current practice as opposed to therapies perceived as separate entities that maybe either complementary, alternative, or both to their current practice. This preference may also be related to the fact that the definition of integrative medicine includes the terms “informed by evidence” since ID practitioners are influenced by data and clinical evidence. Aside from data, the “branding” and social framing of these terms may be important to their incorporation into ID practice. Whatever the reasons, it is important to know that there is a strong preference for term integrative medicine over CAM since it may influence how ID practitioners view certain modalities.

We are not as familiar with how the terms are generally viewed in European, Asian, and other countries of the world as there is no published data on this topic to our knowledge. However, within the international community, there are organizations that use the term integrative medicine such as The Society of Complementary Medicine Research (ISCMR) and The European Congress for integrative medicine [22, 23].

Our study showed that 75% viewed CAM and integrative medicine modalities as useful, with a 72% favorable response for symptom alleviation. Interestingly, our study showed that although about 50% of respondents believed that CAM and integrative medicine modalities derive their benefit from placebo effect, 50% also believed that CAM and integrative medicine modalities could directly affect the disease process and the immune system—especially mind-body modalities and botanical medicine.

Because many CAM and integrative medicine modalities are more suited to outpatient and chronic conditions, we hypothesized that there might be a relationship between the amount of outpatient work and HIV care and favorable responses to CAM and integrative medicine modalities. Our results did not support this since there were no significant associations between practice characteristics and recommendation of CAM and integrative medicine modalities. Nevertheless, our results show that ID physicians believed that CAM and integrative medicine modalities could have a role in the treatment plan for chronic conditions typically treated on an outpatient basis such as HIV and HIV-related complications, chronic Lyme disease, and hepatitis.

The practice of infectious diseases relies heavily on information and data; therefore it is not surprising that ID practitioners are data-driven in their clinical practice. The findings from this study support this, as 68% of respondents reported that data based on clinical research was the most important factor in determining treatment plan. In addition, the most significant factors affecting the recommendation or use of CAM and integrative medicine modalities were all related to lack of adequate information with respect to potential drug interactions, efficacy, and safety.

Other studies have demonstrated a relationship between sex, type of practice, and country of birth and favorable attitudes towards CAM and integrative medicine modalities. Our study did not show any relationships between demographic features and familiarity or recommendation patterns so it seems that typical demographic predictors of and attitudes toward CAM and integrative medicine modalities do not seem to hold for ID physicians.

Limitations to our study include the lower than expected response rates. Although historically response rates are used as a measure of survey quality, there is no proven lower limit for an acceptable response rate [24]. Moreover, some data suggest that response rate may not be as strongly associated with validity as previously believed [24]. It is unclear why our response rate was low, but the reason is probably multifactorial. We did not use a monetary incentive and our survey was fairly lengthy. The low response rate may also represent a lack of interest on the behalf of the survey recipients and a bias in the responders. Therefore our results should be interpreted with caution. Another limitation to our study included a significant difference between the responders and nonresponders with respect to type of practice and transition year. In addition, our study was limited to the USA; therefore the results are not indicative of how ID practitioners in other countries view CAM and integrative medicine modalities.

Strengths of our study included a large random sampling of ID practitioners across the United States. This is the first national survey that provides information on the beliefs and practices of ID practitioners with respect to CAM and integrative medicine modalities and some of the potential barriers to their use in ID practice. In addition, our study also provides information about how terminology of integrative medicine versus CAM may be important to how physicians view these modalities. 

## 5. Conclusion

In conclusion, ID physicians appear to be familiar with and willing to recommend some CAM and integrative medicine modalities. They expressed a marked preference and interest in “Integrative Medicine” over “CAM”. They see a role for CAM and integrative medicine modalities in the management of certain types of chronic infectious diseases and their complications. Data regarding clinical efficacy and safety appear to be the major factors that are important to ID physicians and may represent key areas of development for the integration of CAM and integrative medicine modalities within the practice of infectious diseases.

## Figures and Tables

**Table 1:** Integrative medicine and complementary and alternative medicine categories and modalities.

Category	Modality
Mind-body-based modalities	Hypnosis
	Guided imagery
	Meditation
	Yoga
	Tai Chi

Biologically based modalities	Herbal medicine
	Vitamins and mineral supplementation

Manipulative and body-based modalities	Chiropractic
	Massage
	Acupuncture

Energy-based modalities	Qi gong
	Healing touch
	Reiki
	Therapeutic touch

Whole medical systems	Ayurveda
	Homeopathy
	Traditional Chinese medicine

**Table 2:** Demographics and practice characteristics.

Respondent characteristic^a^	Number (%)
*Age *(*y*) *mean *(*range*)	49 (30–80)
*Gender male *	199 (64)
*Race *(*n* = 317)	
White	218
Black	14
Asian	58
Hispanic/Latino	16
Other	12
*Region *	
Northeast	105 (34)
South	99 (32)
Midwest	55 (18)
West	52 (17)
*Type of degree *(*n* = 313)	
MD	288
Other (MBBS, DO, Other)	25
*ID Board certification *	249 (80)
*Place of birth *(*n* = 261)	
USA	206 (79)
Other	55 (21)

Practice characteristic (*n*)	Number (%)

*Practice setting *(*n* = 295)	
Solo, private	30 (10)
Group, private	66 (22)
Institutional	80 (27)
Academic	119 (40)
*Affiliation with a IM/CAM center (n* = 298)	46 (15)
*Years in Practice *(*n* = 299) *median range *	11–20
*Majority of time spent in *(*n* = 290)	
Research	62 (21)
Patient care	196 (68)
Teaching	9 (3)
*Majority of clinical care in *	
HIV (*n* = 307)	91 (30)
Hepatitis B, C (*n* = 303)	24 (8)
Chronic/recurrent infections (*n* = 302)	81 (27)
Hospital acquired infections (*n* = 304)	147 (48)
Outpatient care (*n* = 302)	104 (34)
ID (*n* = 308)	226 (73)
General internal medicine/hospital medicine (*n* = 299)	36 (12)

^a^Number of respondents is indicated in parenthesis if *n* < or > 311.

**Table 3:** Familiarity with and recommendation of CAM and integrative medicine modality.

Modality	% familiarity (*n* = 311)*	% participants familiar with modality and recommended (no. recommended/total no. of familiar)	% participants familiar with modality and did not recommend
Mind-body-based modalities			
Hypnosis/GI	52	28 (46/163)	72
Meditation	69	45 (97/214)	54
Yoga	74	52 (121/227)	48
Tai Chi	61	26 (50/189)	74
Overall	79	74 (142/192)	41
Biologically based modalities			
Herbal medicine	72	32 (70/219)	68
Vitamin and mineral	83	80 (202/252)	20
Overall	84	84 (211/251)	19
Manipulative and body-based modalities			
Chiropractic	77	33 (77/233)	68
Massage	80	62 (151/247)	37
Acupuncture	79	46 (110/241)	54
Overall	85	76 (190/250)	26
Energy-based modalities			
Qi Gong	17	6 (4/77)	90
HT, reiki, TT	39	16 (20/126)	83
Overall	40	29 (24/84)	81
Whole medical systems			
Ayurveda	26	12 (12/95)	86
Homeopathy	55	8 (14/173)	92
TCM	49	11 (18/158)	89
Overall	64	31 (30/96)	85

*% based on actual responses. >98% of questions had responses.

**Table 4:** Factors influencing the use of CAM and integrative medicine modalities.

Factor (*n* = number of respondents)	Major (%)	Minor or not at all (%)
Drug interactions (*n* = 293)	82	18
Research (*n* = 294)	80	20
Knowledge (*n* = 294)	72	28
Insurance (*n* = 292)	24	76
Cost (*n* = 293)	39	61
Referral base (*n* = 288)	39	61
Professional reputation (*n* = 293)	14	86
Fear of judgment (*n* = 293)	4	96
Regulation oversight (*n* = 294)	69	31
